# Shot through with voices: Dissociation mediates the relationship between varieties of inner speech and auditory hallucination proneness

**DOI:** 10.1016/j.concog.2014.05.010

**Published:** 2014-07

**Authors:** Ben Alderson-Day, Simon McCarthy-Jones, Sarah Bedford, Hannah Collins, Holly Dunne, Chloe Rooke, Charles Fernyhough

**Affiliations:** a Psychology Department, Science Laboratories, Durham University, South Road, Durham DH1 3LE, United Kingdom; b Department of Cognitive Science, Macquarie University, Australian Hearing Hub, 16 University Avenue, NSW 2109, Australia

**Keywords:** Inner speech, Dissociation, Self-esteem, Hallucination, Psychosis, Dialogicality

## Abstract

Inner speech is a commonly experienced but poorly understood phenomenon. The Varieties of Inner Speech Questionnaire (VISQ; McCarthy-Jones & Fernyhough, 2011) assesses four characteristics of inner speech: *dialogicality*, *evaluative*/*motivational* content, *condensation*, and the presence of *other people*. Prior findings have linked anxiety and proneness to auditory hallucinations (AH) to these types of inner speech. This study extends that work by examining how inner speech relates to self-esteem and dissociation, and their combined impact upon AH-proneness. 156 students completed the VISQ and measures of self-esteem, dissociation and AH-proneness. Correlational analyses indicated that evaluative inner speech and other people in inner speech were associated with lower self-esteem and greater frequency of dissociative experiences. Dissociation and VISQ scores, but not self-esteem, predicted AH-proneness. Structural equation modelling supported a mediating role for dissociation between specific components of inner speech (evaluative and other people) and AH-proneness. Implications for the development of “hearing voices” are discussed.

## Highlights

• Inner speech, self-esteem, dissociation and hallucination proneness were examined.
• Self-esteem was linked to inner speech but not hallucination proneness.
• Dissociation mediated links between inner speech and hallucination proneness.

## 1 Introduction

Inner speech – the internal monologue that appears to accompany our daily lives – is an experience that will be familiar to many. Often, inner speech is simply defined as a silent form of speech; for example, Levine, Calvanio, and Popovics (1982) call it the “subjective phenomenon of talking to oneself, of developing an auditory–articulatory image of speech without uttering a sound” (p. 391). But the everyday nature of inner speech hides a wealth of complexity (Hurlburt, Heavey, & Kelsey, 2013). Developmentally, inner speech has been proposed to reflect the internalisation of external dialogue and self-directed private speech, facilitating higher order cognitive skills (Vygotsky, 1987). In adulthood, inner speech supports executive functions (Miyake, Emerson, Padilla, & Ahn, 2004) and facilitates self-evaluation and reflection (Morin & Michaud, 2007). It has also been argued that the form of inner speech in adulthood is shaped by its developmental origins. For example, Fernyhough (2004) has argued that inner speech is inherently dialogic, reflecting the external interpersonal discourse from which it came. Inner speech may also change its form as it develops, becoming syntactically and semantically abbreviated or “condensed”. In this sense, inner speech is likely to be more than simply silent, covert speech. Rather, it is a complex and dynamic activity, and one that needs to be examined in more depth.

Various methods of investigating inner speech exist, including questionnaires (Duncan & Cheyne, 1999; Morin, Uttl, & Hamper, 2011), experience sampling (Hurlburt et al., 2013) and task-based methods (Murray, 1967). However, few have included the various developmental characteristics of inner speech, such as dialogicality and condensation, as well as the functions of inner speech, such as action control (Luria, 1961), in a comprehensive measure of everyday experience. One exception is the Varieties of Inner Speech Questionnaire (VISQ; McCarthy-Jones & Fernyhough, 2011), a scale that measures self-reported inner speech along four distinct dimensions: (i) *dialogicality*, the communicative and conversational quality of inner speech; (ii) *condensation*, or the extent to which inner speech is syntactically and semantically abbreviated, (iii) *evaluative*/*motivational* inner speech, such as saying “I should do this” to oneself, and (iv) the presence of *other people*’*s* voices in inner speech.

An initial validation of the VISQ (McCarthy-Jones & Fernyhough, 2011) demonstrated two advantages of this approach. First, the VISQ can provide a more nuanced picture of the range of ways in which inner speech is used and experienced; in data from a student sample, dialogic and evaluative characteristics of inner speech appeared to be very common, while a substantial minority reported experiences of condensation and other people’s voices in their inner speech (McCarthy-Jones & Fernyhough, 2011). Secondly, given that inner speech is proposed to be the raw material of some auditory verbal hallucinations (‘hearing voices’) (Bentall, 1990; Frith, 1992; Shergill et al., 2003), and may play a causal or maintenance role in anxiety and depression (Harrington & Blankenship, 2002), the VISQ can be used to examine how specific types of inner speech may be related to psychopathological processes. In their original study, McCarthy-Jones and Fernyhough found that self-reported evaluative inner speech and the presence of other people in inner speech were positively related to anxiety. Alongside this, proneness to auditory hallucinations (AH) had bivariate correlations with evaluative, other people and dialogic inner speech, although after controlling for potentially confounding factors, only dialogic characteristics remained a significant predictor of AH-proneness (McCarthy-Jones & Fernyhough, 2011).

The present study sought to extend this work by examining the interrelations between inner speech and two other constructs that have been related to the development and experience of psychosis-like phenomena: self-esteem (Krabbendam et al., 2002) and dissociation (Altman, Collins, & Mundy, 1997).

Self-esteem refers to the set of positive and negative beliefs and feelings that people have about themselves. It has been related to the severity and content of hallucinations in clinical studies (Romm et al., 2011; Smith et al., 2006) and has been proposed to shape the interactions people have with the voices they hear (Paulik, 2012). In work with the general population, self-esteem has been observed to predict later development of psychosis (Krabbendam et al., 2002), and has been associated with increased hallucination-proneness (Gaweda, Holas, & Kokoszka, 2012; Gracie et al., 2007; Pickering, Simpson, & Bentall, 2008).

Dissociation refers to a “lack of normal integration of thoughts, feelings and experiences into the stream of consciousness and memory” (Bernstein & Putnam, 1986, p. 727), and is typified by feelings of depersonalization, derealisation and absorption. Diagnostic overlaps between dissociative and psychotic disorders have often been noted (Allen & Coyne, 1995), and dissociation is strongly related to rates of psychotic experiences, including hallucinations (Altman et al., 1997; Glicksohn & Barrett, 2003; Kilcommons & Morrison, 2005; Merckelbach, Rassin, & Muris, 2000). More recently, specific links between dissociation and voice-hearing have been proposed (Moskowitz & Corstens, 2008), with dissociative experiences potentially playing a predisposing role or acting as a preliminary stage in the development of AHs (Perona-Garcelán et al., 2011; Varese, Barkus, & Bentall, 2012).

In addition to their documented association with hallucinations, there are *prima facie* reasons to think that both self-esteem and dissociation might overlap with specific aspects of inner speech. Given its proposed role in self-assessment and reflection, evaluative aspects of inner speech would appear relevant to an individual’s self-concept and social rank. The presence of other voices in inner speech, in contrast, has a phenomenological similarity with dissociative experiences of depersonalization and detachment; a separation of the self and other. This can be seen in example items from the VISQ, such as “I experience the voices of other people asking me questions in my head” (McCarthy-Jones & Fernyhough, 2011). As such, the first aim of this study was to establish what links, if any, there are between varieties of inner speech, self-esteem and dissociative traits. We hypothesised that evaluative inner speech scores in particular would be strongly associated with self-esteem, while other people in inner speech would be closely associated with dissociation scores.

The second aim of this study was to investigate how any potential relations between the varieties of inner speech, dissociation and self-esteem might influence the previously documented association between inner speech and AH-proneness (McCarthy-Jones & Fernyhough, 2011). If self-esteem and dissociation were to be found to be related to both inner speech and AH-proneness, then they could represent potential confounding variables, or act as either mediators or moderators of the link between inner speech and AH-proneness. Dissociation in particular has been proposed to act as a mediator in the development of hallucinations. In patients with schizophrenia spectrum disorders, dissociative traits positively mediate the effects of childhood trauma (Varese et al., 2012), and attentional focus (Perona-Garcelán et al., 2011) on hallucination proneness.

As it is currently unclear how inner speech may become transmuted into AHs, an examination of whether dissociation and/or self-esteem are potential mediators is of considerable potential value. This was accomplished by testing the fit of a number of potential models of the relation between self-esteem, dissociation and the varieties of inner speech using structural equation modelling.

## 2 Materials and methods

### 2.1 Participants

A sample of 156 students (22 male) was recruited from a United Kingdom university. The mean age was 20.02 years (SD = 2.11, range 18–31). Participants were invited to take part via a web advert and received course credit for participation. Participants indicated their age and gender and provided an email address if they wished to be contacted for a follow-up study (which is not reported here). All procedures were approved by the local university ethics committee.

### 2.2 Measures

#### 2.2.1 Varieties of Inner Speech Questionnaire (VISQ; McCarthy-Jones & Fernyhough, 2011)

The Varieties of Inner Speech Questionnaire is an 18-item self-report scale measuring four dimensions of inner speech. Participants endorse items about their experience of inner speech in relation to *dialogicality* (“I talk back and forward to myself in my mind about things”), *evaluative and motivational* characteristics (“I talk silently to myself telling myself not to do things”), *condensation* (“I think to myself in words using brief phrases and single words rather than full sentences”) and the presence of *other voices* (“I experience the voices of other people asking questions in my head”). Responses are made on a 6-point Likert scale ranging from “Certainly does not apply to me” (1) to “Certainly applies to me” (6). Each subscale of the VISQ has high internal reliability (Cronbach’s *α* > .80) and moderate to high test–retest reliability (>.60).

#### 2.2.2 Rosenberg Self-Esteem Scale (RSES; Rosenberg, 1965)

The Rosenberg Self-Esteem Scale is a widely used 10-item measure of global self-esteem with high test–retest and internal reliability (*α* = .8–.9; Fleming & Courtney, 1984; Robins, Hendin, & Trzesniewski, 2001). Respondents use a four-point scale (strongly agree, agree, disagree and strongly disagree) to classify evaluative statements about themselves, such as “On the whole, I am satisfied with myself”.

#### 2.2.3 Dissociative Experiences Scale – Second Revision (DES-II; Carlson & Putnam, 1993)

The Dissociative Experiences Scale measures frequency of dissociative experiences on a 28-item scale. Participants indicate what percentage of the time they experience dissociative states ranging from 0% to 100%, e.g., “Some people have the experience of finding themselves in a place and have no idea how they got there. What percentage of the time does this happen to you?”. The original DES has been shown to have good reliability and validity; test–retest reliabilities range from .7 to .9 (Carlson & Putnam, 1993) with a mean internal reliability of .93 (Van IJzendoorn & Schuengel, 1996). The DES-II differs only in the use of incremental response options rather than a visual analogue scale.

#### 2.2.4 Revised Launay–Slade Hallucination Scale: Auditory Subscale (LSHS-R; Morrison, Wells, & Nothard, 2000)

The Launay–Slade Hallucination Scale is a commonly used measure of hallucination proneness in non-clinical samples. The revision used here (Morrison et al., 2000) was developed specifically to separate factors relating to auditory and visual hallucination proneness. Five items relating specifically to auditory hallucination experiences (e.g., “I have had the experience of hearing a person’s voice and then found that there was no one there”) were used here following their use in McCarthy-Jones and Fernyhough (2011), in which they were observed to have good internal reliability (Cronbach’s *α* = .73). Items were scored on four-point scale ranging from “Never” (1) to “Almost Always” (4).

### 2.3 Data analysis

Relationships between the VISQ and other variables were first analysed using Pearson’s product moment correlation co-efficients. Secondly, hierarchical regression was used to assess the ability of inner speech, self-esteem and dissociation to predict hallucination proneness, with LSHS-R scores acting as the dependent variable. Structural equation modelling (SEM) was then used to test competing models of the relationship between the variables. All analyses were conducted in SPSS 20 and AMOS 7.0. Unless otherwise noted, all Durbin–Watson statistics and parameters regarding collinearity and normality of residuals were within acceptable bounds.

A number of arguments have been made as to the most appropriate way in which to correct alpha to control for the altered experiment-wise error rate resulting from undertaking multiple comparisons. For example, it has been argued that a different approach is needed for exploratory and confirmatory studies (Bender & Lange, 2001). For confirmatory studies, Bonferroni corrections are generally viewed as appropriate (Bender & Lange, 2001). Thus, for the part of this study which involved confirmatory tests, i.e., which repeated the analyses of McCarthy-Jones and Fernyhough (2011) by examining four bivariate correlations (between the four VISQ subscales and AH-proneness), a Bonferroni corrected alpha was employed of *α*′ = .05/4 = .0125. For all other analyses, which were exploratory, *α* was retained at .05. This is in accordance with Perneger, 1998 argument that “simply describing what was done and why, and discussing the possible interpretations of each result, should enable the reader to reach a reasonable conclusion without the help of Bonferroni adjustments” (p. 1237).

## 3 Results

### 3.1 Descriptive statistics

Table 1 displays descriptive statistics for the four dimensions of the VISQ, as well as the RSES, DES and LSHS-R. Mean scores were highest for evaluative inner speech and dialogic inner speech, followed by other people in inner speech and condensed inner speech. If the presence of specific types of inner speech was defined as positive endorsement of the majority of items on each subscale (i.e., a response of 4 or higher), 80.1% of participants reported dialogic inner speech, 87.2% reported evaluative inner speech, 22.4% reported other people in inner speech, and 28.2% reported condensed inner speech. Internal reliability scores (Cronbach’s *α*) were good for each subscale (all >.70). Both mean scores and reliability co-efficients were comparable to the original VISQ data reported by McCarthy-Jones and Fernyhough (2011). Reliability for self-esteem and dissociation scores was high (*α* > .90). Cronbach’s *α* was lower for hallucination-proneness (0.69) although still well within the acceptable range (i.e., >0.6).

### 3.2 Correlations of inner speech with self-esteem, dissociation and auditory hallucination-proneness

Table 2 shows bivariate correlations among VISQ, RSES, DES and LSHS-R scores. Evaluative inner speech (*p* = .011) and other people in inner speech (*p* = .022) scores were significantly negatively correlated with global self-esteem scores, such that higher self-esteem was associated with less evaluative inner speech and fewer instances of other people in inner speech. Both other people and evaluative inner speech were also positively related to frequency of dissociative experiences (see table 2). AH-proneness was positively associated with dialogic inner speech (*p* < .001), evaluative inner speech (*p* < .001) and other people in inner speech (*p* < .001).

### 3.3 Predicting proneness to auditory hallucinations

A hierarchical multiple linear regression was performed to assess the unique contribution of inner speech (VISQ), self-esteem (RSES) and dissociation (DES) in predicting auditory hallucination proneness. Age and gender were included in the first block as control variables. This was then followed by the four VISQ subscales in the second block (to replicate the findings from McCarthy-Jones & Fernyhough, 2011), RSES in the third block, and DES in the fourth block.

All residuals were normal and measures of multicollinearity were in an acceptable range. While the first block was non-significant (*p* > .100), each of the second, *F* (6, 149) = 6.56, *p* < .001, third, *F* (7, 148) = 5.59, *p* < .001, and fourth, *F* (5, 214) = 8.67, *p* < .001, blocks significantly predicted AH-proneness. In Block 2 (*R*^2^ = .21), other people in inner speech, *β* = .250, *p* = .001, and evaluative inner speech, *β* = .198, *p* = .023, were the only significant predictors. This was also the case in Block 3 (*R*^2^ = .21); the addition of self-esteem made no significant change to the model, Δ*R*^2^ = .00, *p* = .873, and self-esteem failed to predict AH-proneness, *β* = −.012, *p* = .873.

In contrast, the addition of DES scores in Block 4 significantly improved the model (ΔR^2^ = .11, *p* < .001, *R*^2^ = .32). Dissociation scores significantly predicted AH-proneness, *β* = .367, *p* < .001, while VISQ predictors were only evident at the level of trends for other people in inner speech, *β* = .134, *p* = .078, and condensed inner speech, *β* = −.130, *p* = .066. Thus, the predictive value of VISQ subscales was almost completely removed with the inclusion of dissociation scores.

### 3.4 Mediation and moderation of AH-proneness

#### 3.4.1 Mediator effects

**Fig. 1:**
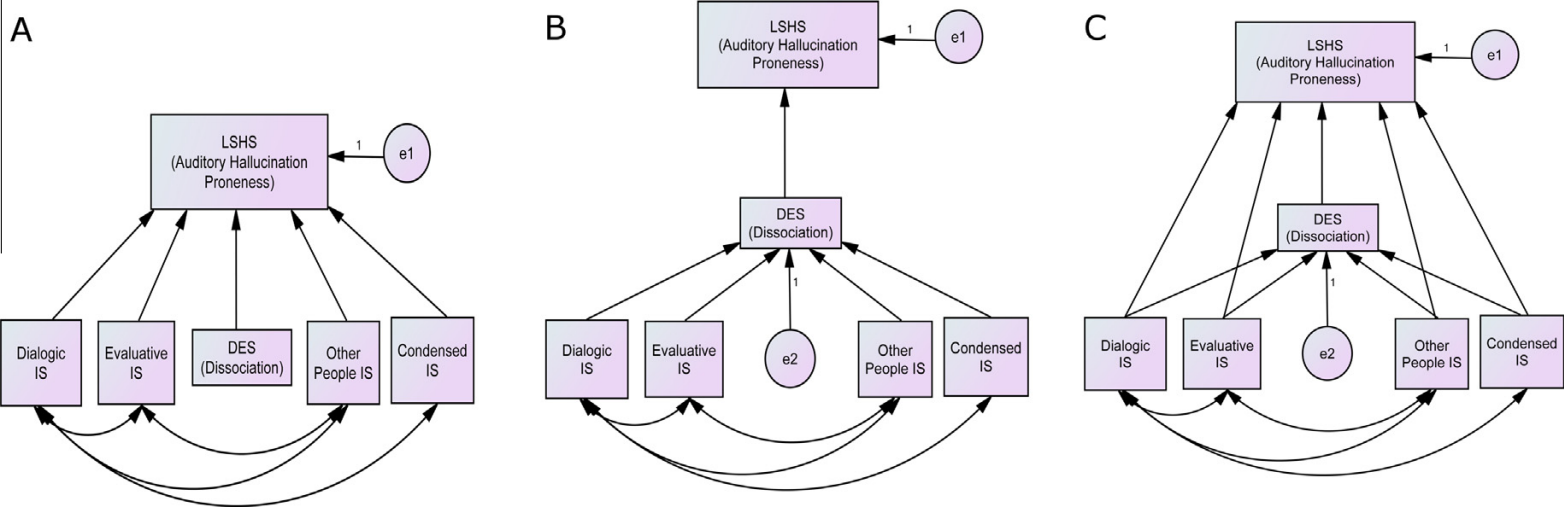
Three models of relations between inner speech, dissociation and auditory hallucination proneness to be tested.

Structural equation modelling (SEM) in AMOS was used to further examine the relationship between inner speech, dissociation and AH-proneness. As the regression analyses showed self-esteem not to predict AH-proneness, this was excluded from the SEM. To allow for non-normal data distributions, an asymptotically free distribution (ADF) method was used combined with a Yuan–Bentler statistic, which is appropriate for sample sizes less than 200 (Yuan & Bentler, 1999). Three models were compared: (a) dissociation and inner speech scores as independent predictors of AH-proneness, (b) dissociation as a full mediator of the relationship between inner speech and AH-proneness, and (c) dissociation as a partial mediator of this relationship (see Fig. 1). Each model also contained covariate links between the VISQ domains where these were suggested by bivariate correlations.

The first model represented a situation in which dissociation and inner speech present unique pathways towards increased hallucination proneness. The latter two models tested whether dissociation plays a role in mediating the relationship between inner speech and hallucination proneness, either by fully accounting for their covariance (full mediation) or only accounting for some of it (partial mediation). If dissociation was observed to fully mediate this relationship, inner speech could only be said to have an indirect effect on AH-proneness.

Of the three models, only Model *C* (partial mediation) provided an adequate fit to the data, *X*^2^_ADF_ (2) = 1.51, *p* = .471, *T_F_* (2, 154) = 0.75, *p* = .475, RMSEA = .000, GFI = .997, CFI = 1.00. Both Model *A*, *X*^2^_ADF_ (6) = 20.69, *p* = .002, *T_F_* (6, 150) = 3.34, *p* = .004, RMSEA = .126, GFI = .962, CFI = .737, and Model *B*, *X*^2^_ADF_ (6) = 16.47, *p* = .011, *T_F_* (6, 150) = 2.67, *p* = .017, RMSEA = .106, GFI = .969, CFI = .813 were significantly different from the data, indicating an inadequate fit.

Bootstrap statistics were used to assess the significance of direct and indirect effects on AH-proneness. Direct effects on AH-proneness were observed for dissociation, *p* = .004, other people in inner speech, *p* = .042, and condensed inner speech, *p* = .025. A marginal but non-significant direct effect was also evident for evaluative inner speech, *p* = .063. Indirect effects on LSHS, via dissociation, were observed for other people in inner speech, *p* = .004 and evaluative inner speech, *p* = .006, but not condensed inner speech, *p* = .615. No direct or indirect effects were observed for dialogic inner speech (all *p* > .200).

**Fig. 2:**
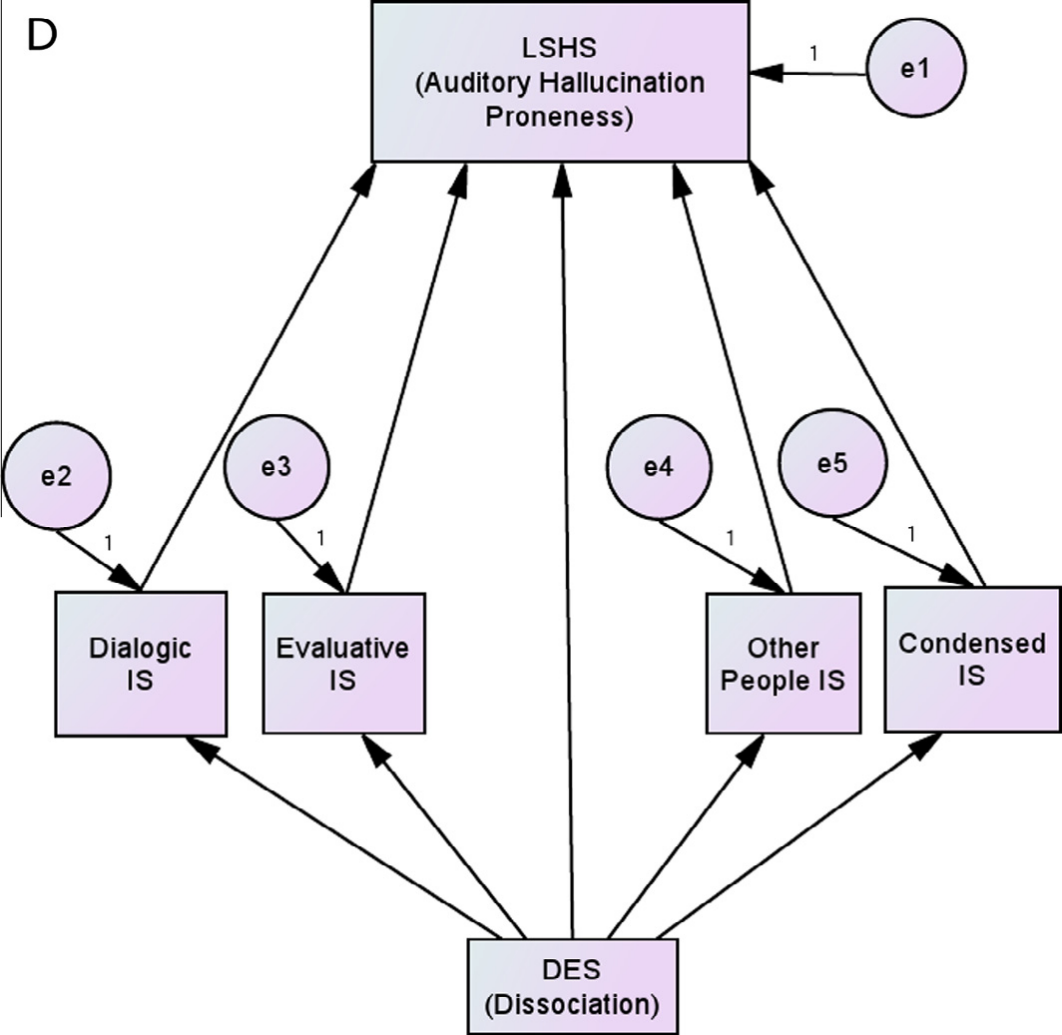
Model in which inner speech characteristics mediate the relationship between dissociation and auditory hallucination proneness.

To further test the specific direction of these relationships, a model, *D*, was constructed that matched Model *C* but included VISQ scores as mediating variables and DES as their predictor (see Fig. 2). In such a scenario, dissociation would be associated with different characteristics of inner speech, and these in turn would lead to greater hallucination-proneness. However, this did not produce an adequate fit to the data, *X*^2^_ADF_ (6) = 24.81, *p* < .001, *T_F_* (6, 150) = 4.00, *p* < .001, RMSEA = .142, GFI = .934, CFI = 0.664.

#### 3.4.2 Moderator effects

To examine whether dissociation might moderate rather than mediate the effect of inner speech scores on hallucination proneness, a multiple linear regression was performed using the same predictors as above, plus mean-centred interaction terms between DES scores and the four VISQ subscales. This, however, yielded no new significant predictors of auditory hallucination proneness (all interaction effects *p* > .70, *β* < .01).

### 3.5 Predictive relationships with overlapping items removed

Finally, while aimed at nominally separable constructs, measures of inner speech, hallucination-proneness and dissociation can sometimes be very similar in the kinds of experiences they describe. It is therefore important to rule out any trivial relationships between these constructs that might be caused by individual items that happen to overlap. To address this, we reran each of our main analyses with subsets of items removed from our measures of inner speech and dissociation.

A subset of items was removed from the VISQ and the DES (the RSES had little overlap with other scales). In the VISQ, two items explicitly refer to hearing other people’s “actual voices”; item 12 “I hear other people’s actual voices in my head, saying things they have never said to me before” and item 16 “I hear other people’s actual voices in my head, saying things that they actually once said to me”. Both these items were removed from the “Other People” subscale to avoid trivial overlaps with the LSHS-R. In the DES, two items were removed; one clearly overlapped with hallucination proneness and inner speech (item 27: “Some people sometimes find that they hear voices inside their head that tell them to do things or comment on things that they are doing.”) while another was deemed to be similar to questions about inner speech (item 21: “Some people sometimes find that when they are alone they talk out loud to themselves”).

Each of the above analyses (correlations, regression and SEM) was rerun using the adjusted scores for other people in inner speech and dissociation. The new scores for other people in inner speech significantly correlated with LSHS-R (*r* = .24). RSES (*r* = −.21) and the adjusted DES (*r* = .31; all *p* < .05), albeit to a lesser degree than beforehand. When the new DES and other people VISQ scores were entered into the hierarchical regression, the results for each model were almost identical to those observed originally, with DES the single significant predictor in the final model (*β* = .382, *p* < .001). The adjusted score for other people in inner speech was a significant predictor in blocks 2 and 3, but was no longer evident (even at trend level) in the final model (*p* = .406).

Re-analysis using SEM also produced similar results. When the adjusted scores for DES and other people in inner speech were included, only Model *C* yielded an appropriate fit to the data, *X*^2^_ADF_ (2) = 2.79, *p* = .247, *T_F_* (2, 154) = 1.39, *p* = .253, RMSEA = .051, GFI = .995, CFI = .985. Bootstrap analysis yielded similar direct effects for DES (*p* = .004) and condensed inner speech (*p* = .033) on hallucination proneness, alongside a new direct effect for evaluative inner speech (*p* = .028). Indirect effects were evident for evaluative inner speech (*p* = .007) and the adjusted other people in inner speech (*p* = .008). Thus, removal of overlapping items (i) made little difference to the role of dissociation, (ii) reduced the role of other people in inner speech, but also (iii) highlighted a greater contribution of evaluative inner speech.

## 4 Discussion

The present study examined the relationships among inner speech, self-esteem and dissociation, and how these factors combine to predict proneness to auditory hallucinations. The main finding was that dissociation mediated the relationship between inner speech quality and the tendency to experience auditory hallucinations. Specifically, dissociation mediated the effect of other people in inner speech and evaluative/motivational inner speech on auditory hallucination proneness. There was also some evidence to suggest direct effects of evaluative inner speech and condensed inner speech on proneness to auditory hallucinations. Correlational analysis indicated a relationship between self-esteem, evaluative/motivational inner speech and other people in inner speech, but no relationship was evident between self-esteem and hallucination proneness.

Consistent with prior findings (McCarthy-Jones & Fernyhough, 2011), the four VISQ subscales showed good reliability and specific relationships with proneness to auditory hallucinations; dialogic and evaluative characteristics of inner speech and the presence of other voices in inner speech positively correlated with AH-proneness. However, although McCarthy-Jones and Fernyhough found dialogic inner speech to account for unique variance in auditory hallucination proneness (after controlling for all VISQ subscales, age, gender, anxiety, depression and visual hallucination-proneness), in this dataset it did not account for any unique variance in AH-proneness, at any stage of the regression or SEM analysis. Possible explanations for this are the collinearity of this particular domain of inner speech with other aspects of inner speech, and the use of different control variables (anxiety and depression) in McCarthy-Jones and Fernyhough’s original study. Given that evaluative inner speech and other people in inner speech correlate significantly with anxiety (McCarthy-Jones & Fernyhough, 2011), it may be that dialogicality is only predictive of hallucination-proneness once factors relating to anxiety are partialled out. Replication data are needed to clearly delineate which aspects of inner speech are the best predictors of AH-proneness and what role dialogic characteristics may play, if any.

Dissociative traits were most strongly associated with the presence of other people’s voices in inner speech, although they were also linked to evaluative inner speech. This suggests that a greater tendency to experience other voices in everyday thinking may in itself be a dissociative tendency or could be a precursor of more developed dissociative states such as depersonalisation and identity confusion (Steinberg, 1991). When dissociation scores were included in the model for predicting AH-proneness, they accounted for some, but not all, of the predictive power of inner speech. The SEM analysis suggested that the best fit to the data involved a model where DES partially mediated the link between different subscales of the VISQ and hallucinations. Importantly, this was true even when items with potentially overlapping content were removed from the DES and VISQ, and highlighted a range of direct and indirect effects of inner speech, with other people in inner speech being fully mediated, evaluative inner speech partially mediated, and condensed inner speech appearing to show a small but unmediated effect. Given that some of the direct effects were only evident in the SEM and not in the hierarchical regression (particularly concerning condensed inner speech), these relations should be interpreted with caution and require further replication. Nevertheless, it appears that different characteristics of inner speech may be picking out independent and alternative routes to AH-proneness.

These data support the role of dissociation as a mediating mechanism in the development of hallucinatory experiences. Varese et al. (2012) observed DES scores to positively mediate the effect of childhood trauma on hallucination-proneness in hallucinating patients. This finding has been replicated in a non-clinical university sample using alternative measures of two specific dissociative experiences, depersonalisation and absorption (Perona-Garcelán et al., in press). While such findings point towards a specific role for dissociation in the link between trauma and hallucination, the present data may suggest a wider mediating role for dissociative traits in the development of unusual perceptual experiences, as seen for example in data on attentional focus and hallucinations (Perona-Garcelán et al., 2011). In such a scenario, characteristics of inner speech could develop into hallucination via a dissociative stage, or there may be an additive effect between inner speech and dissociative traits that gives rise to hallucination proneness. Given that we did not observe any significant moderator effects in this dataset, these results appear to support the former scenario over the latter.

The converse could of course be possible; it may be that having a tendency towards dissociation could lead to the development of specific kinds of inner speech – such as talking in other voices – and this in turn leads to the development of hallucinations. When this scenario was modelled with the VISQ scores as mediators (Model *D*) it did not produce an adequate fit to the data, which would suggest that inner speech does not play such a role. However, only longitudinal data on inner speech, dissociation and hallucination proneness could convincingly split apart these possibilities. Yet to be examined also are the potential relationships between earlier childhood experiences (including trauma and other adversity) and characteristics of inner speech. A tentative hypothesis would be that the presence of other voices and evaluative characteristics of inner speech are likely to have strong developmental roots, both based on their theoretical origin (Fernyhough, 2004) and links between early adversity and later hallucination proneness (Bentall, Wickham, Shevlin, & Varese, 2012).

This is the first study to demonstrate links between specific varieties of inner speech and self-esteem. As predicted, a greater tendency to engage in evaluative forms of inner speech (“I talk silently to myself, telling myself not to do things”) was associated with lower self-esteem. Though such an association cannot demonstrate causality, it seems plausible to suggest that inner speech with a strongly normative and ruminative component could have a negative impact on self-concept and social rank. Equally, the former could be an expression of the latter, if lower self-esteem was taken to lead to more frequent evaluation and rumination in inner speech. In either case, this contrasts with the suggestion that evaluative aspects of inner speech generally play a positive role in enabling self-awareness and self-reflection (Morin & Everett, 1990); an increase in such tendencies could equally be associated with over-analysis and self-criticism. It is important here to consider the valence of the kind of inner speech that is occurring; some evaluations will be more positive (e.g., “I *can* do this”) and others negative (“I really *shouldn*’*t* have done that…”) and this will vary with context. The items in the VISQ are designed to be balanced in their appraisals, but further study with a greater range of positive and negative items is really needed to clarify the relationship between self-concept and inner speech use. Self-esteem was also negatively correlated with reports of other people in inner speech, indicating that experiencing other people’s voices in everyday verbal thinking is linked to lower self-esteem and a more negative self-concept. The lack of any relationship between self-esteem scores and AH-proneness contrasts with prior reports of self-esteem effects (Pickering et al., 2008) but is consistent with prior null findings in a similar population (Jones & Fernyhough, 2007).

There are a range of caveats to be acknowledged in interpreting these results. First, these data come from a non-clinical, university sample and only indicate relationships between general population traits associated with psychosis. The correlations observed are broadly consistent with those reported in clinical studies (as in, for example, the case of dissociation and hallucination; Kilcommons & Morrison, 2005) but any extrapolation to clinical populations must be made with caution. Second, the present results are purely correlational and do not provide evidence of causal relationships between the data. Time-based data via longitudinal studies or use of experience sampling methods (ESM; Csikszentmihalyi & Larson, 1987) are required to assess causal relationships between these variables.

Third, these data are reliant on self-reports for the measurement of experiences that may be hard to report reliably and accurately. Previously used inner speech questionnaires (although not the VISQ) have been criticised for showing a lack of convergent validity (Uttl, Morin, & Hamper, 2011) and it has been suggested that self-reported inner speech is likely to be overestimated in general questionnaires (Hurlburt et al., 2013). Similarly, the validity and reliability of self-reports for relatively infrequent, unusual phenomena such as hearing a voice or having a dissociative experience is hard to gauge in non-clinical samples, and the possibility of over-reporting cannot be ruled out. The mean DES score observed here, although comparable with other student/college studies (e.g., Merckelbach, Muris, Rassin, and Horselenberg, 2000) was relatively high, suggesting that some overestimation may have occurred. The internal reliability of hallucination-proneness scores, though in the adequate range, was also lower than the other measures used (*α* = .69). An alternative approach would be to use more items to examine hallucination-proneness, although that may be at the risk of losing specificity, as existing longer scales often conflate auditory experiences with visual hallucinations and other unusual phenomena (Morrison et al., 2000) The use of ESM is equally important here to examine whether day-to-day, random sampling of such experiences actually corroborates participants’ general trait reports. Alternatively, use of questionnaires and ESM could be combined with electromyographical techniques to assess covert muscle activity associated with inner speech (see, for example, Rapin, Dohen, Polosan, Perrier, & Loevenbruck, 2013).

Finally, while the VISQ is an explicitly multi-dimensional scale, the measures used here for self-esteem, dissociation and hallucination-proneness represent unidimensional indices of complex phenomena. As the first study to investigate this group of constructs in relation to inner speech, we selected measures partly for simplicity and to limit the number of potential comparisons examined. In the case of hallucinatory experiences, auditory hallucinations were focused on here rather than visual experiences or other modalities because of the putative association of AH with inner speech (Fernyhough, 2004) and their proposed links to dissociation (Moskowitz & Corstens, 2008). Nevertheless, an important focus for future studies would be an assessment of the relationships among VISQ dimensions and separable aspects of dissociation (such as depersonalisation and absorption), and the specificity of such relationships for understanding auditory hallucinations.

## 5 Conclusions

In conclusion, these results highlight the importance of asking about a variety of experiences in inner speech. Different characteristics of inner speech relate to self-esteem, dissociation and AH-proneness in specific ways that would otherwise be missed by a less fine-grained analysis of how people talk to themselves. Further examination of these characteristics will shed light on how ordinary characteristics of day-to-day inner experience can, in some cases, become the extraordinary.

## Figures and Tables

**Table 1:** Descriptive statistics for inner speech, AH-proneness, self-esteem and dissociation.

	Present study			McCarthy-Jones and Fernyhough (2011)		
	*M*	(SD, range)	*α*	*M*	(SD, range)	*α*
VISQ dialogic	18.27	(4.12, 4–24)	0.77	17.80	(4.67, 5–24)	0.83
VISQ evaluative/motivational	19.39	(3.57, 5–24)	0.71	18.68	(4.31, 4–24)	0.80
VISQ other people	12.42	(5.79, 5–30)	0.79	11.99	(6.66, 5–30)	0.88
VISQ condensed	14.24	(5.22, 5–27)	0.79	15.09	(5.60, 5–28)	0.80
AH-proneness (LSHS-R: AH)	9.13	(2.67, 5–16)	0.69	9.46	(3.02, 5–20)	0.73
Self-esteem (RSES)	18.60	(5.17, 2–30)	0.92	–	–	–
Dissociation (DES)	21.28	(15.48, 1–69)	0.95	–	–	–

*Note: α* = Cronbach’s alpha; AH = auditory hallucination, VISQ = Varieties of Inner Speech Questionnaire.

**Table 2:** Correlations between inner speech, AH-proneness, self-esteem and dissociation.

VISQ	Evaluative	Other people	Condensed	Self-esteem	Dissociation	AH-proneness	
						Present study	McCarthy-Jones and Fernyhough (2011)
Dialogic	.52⁎⁎⁎	.21⁎⁎	−.16	−.10	.17	.29⁎⁎⁎	.32⁎⁎⁎
Evaluative		.20⁎	−.09	−.20⁎	.26⁎⁎	.31⁎⁎⁎	.29⁎⁎⁎
Other people			.02	−.18⁎	.34⁎⁎⁎	.32⁎⁎⁎	.31⁎⁎⁎
Condensed				−.02	.04	−.18	.05

VISQ = Varieties of Inner Speech Questionnaire, AH = auditory hallucination.

⁎ *p* < .05.

⁎⁎ *p* < .01.

⁎⁎⁎ *p* < .001.
